# From fragmentation to empowerment: community rehabilitation care and health literacy as drivers of integrated care

**DOI:** 10.3389/frhs.2026.1778931

**Published:** 2026-03-11

**Authors:** Ana Catarina Maia, Andreia Félix

**Affiliations:** 1 University Coimbra, UICISA:E, ESEUC, Coimbra, Portugal; 2 Nursing Research Innovation and Development Centre of Lisbon (CIDNUR), School of Nursing, University of Lisbon, Lisbon, Portugal; 3 University Coimbra, ESEUC, Coimbra, Portugal

**Keywords:** community rehabilitation, digital health, health equity, health literacy, integrated care, person-centered care

## Abstract

In the 21st century, rehabilitation care in community contexts has emerged as a strategic structuring axis for more equitable, sustainable and person-centred health systems. Rather than being merely a therapeutic addition, rehabilitation is now widely recognised as a fundamental right and a valuable social investment that promotes functionality, engagement, participation and quality of life throughout life. Nevertheless, the persistence of hospital-centred care models, fragmentation between levels of care and unequal access continue to compromise the continuity and effectiveness of interventions. This perspective puts forward a new conceptual idea by presenting community rehabilitation, health literacy and digital transformation as an interconnected triad that facilitates the shift from fragmented care pathways to integrated, empowerment-oriented systems. We propose a conceptual framework that positions community-based rehabilitation as a longitudinal process embedded in everyday contexts; health literacy as a driver of co-responsibility and equity; and digital transformation as enabling infrastructure for coordination and continuity. The implications of this model extend to policy development, service organisation and professional practice, emphasising the necessity of governance arrangements, workforce competencies and digital strategies that can support truly person-centred, integrated care.

## 1 Introduction

For decades, rehabilitation care was perceived as a complementary stage in the healthcare continuum, a process to be initiated only after medical care. However, this reductionist view has been progressively replaced by a broader understanding that recognizes rehabilitation care as an essential pillar of universal health coverage, alongside promotion, prevention, and treatment.

The World Health Organization (WHO), through its initiative Rehabilitation 2030: A Call for Action (1, 2), has reinforced the need to reposition rehabilitation as a cross-cutting priority in public policy, indispensable to the sustainability, equity and resilience of health systems.

The global evidence is unequivocal: an estimated 2.41 billion people worldwide need some form of rehabilitation, about one in three individuals (3). This number has been increasing significantly, driven by population ageing and the growing prevalence of disabling chronic diseases, such as cardiovascular, respiratory, neurological and musculoskeletal (2, 4). The United Nation predicts that by 2050, the number of people over 60 will double, exceeding two billion — a scenario that will place increased pressure on health systems and the need for integrated rehabilitation responses (5).

The COVID-19 pandemic has intensified and highlighted these needs, revealing persistent sequelae, such as fatigue, dyspnea and cognitive deficits, that require structured, continuous and community-based responses (6). At the same time, the disruption and overload of services during lockdowns exposed the fragility of existing rehabilitation networks and the absence of continuity of care plans, confirming the urgency of more decentralized, collaborative and technologically supported models (7).

In Europe, several strategic documents, notably Health at a Glance: Europe 2022 (8) and State of Health in the EU: Companion Report (9) highlight the need for a transition from hospital-centric models to integrated, community-based approaches guided by principles of proximity and participation. In Portugal, although the National Health Plan and the National Network for Integrated Continuing Care recognize the structural role of rehabilitation, territorial asymmetries, disarticulation between levels of care and persistent underfunding persist (10).

It is in this context that rehabilitation care in a community setting takes on new meaning, not only as a therapeutic strategy, but as an instrument of citizenship and social cohesion. By ensuring functionality, participation and autonomy, it simultaneously responds to clinical, social and economic challenges. At the same time, health literacy emerges as an essential condition for empowerment and co-responsibility, enabling individuals to understand, manage and actively participate in decisions regarding their rehabilitation process (11, 12).

This Perspective makes a distinct conceptual contribution by explicitly articulating community rehabilitation, health literacy and digital transformation as an interdependent triad rather than as separate components of integrated care. While existing integrated care and rehabilitation frameworks emphasise coordination across levels of care, this perspective argues that effective integration requires functional support in community settings to be aligned, individuals and communities to have the capacity to engage meaningfully with care, and digital infrastructures to enable continuity, communication, and shared responsibility, all simultaneously. By presenting community rehabilitation as a driver of empowerment, social cohesion, and equity, this triadic model redefines rehabilitation as not just a service configuration, but also a structuring strategy for resilient, person-centred health systems.

## 2 Rehabilitation care in transition: from hospital-based to community-based rehabilitation

Rehabilitation care has become a central component of resilient and equitable health systems in response to population ageing, increasing multimorbidity and improved survival following acute illness. These trends have exposed the limitations of hospital-centred and episodic rehabilitation approaches, reinforcing the need to conceptualise rehabilitation as a longitudinal process that accompanies individuals across settings and stages of the life course (13, 14).

In this context, the WHO has progressively repositioned rehabilitation as an essential health service and a core pillar of universal health coverage. Current WHO frameworks emphasise that rehabilitation should be integrated across the continuum of care, including hospital services, primary health care, community-based services and long-term care, rather than being confined to hospital episodes (2, 15). Rehabilitation focuses on individuals' capacities rather than on disease, aiming to reduce disability with interaction with their environment and to maximize their ability to live, work, and learn to their full potential. This shift aligns rehabilitation with broader public health goals related to healthy ageing, equity and health system sustainability (1).

### 2.1 Fragmentation, inequities and the limits of hospital-centred rehabilitation

Despite increasing policy recognition, rehabilitation care across Europe remains fragmented. Insufficient coordination between hospital services, primary health care and community-based resources frequently results in poorly planned discharges, interrupted rehabilitation trajectories and avoidable hospital readmissions (16, 17).

These discontinuities disproportionately affect people living with chronic conditions and long-term functional limitations, for whom continuity of rehabilitation is essential to prevent functional decline and dependency (18).

Fragmentation is further compounded by territorial and organisational inequities. Significant unmet rehabilitation needs persist, particularly in rural and socioeconomically disadvantaged areas, reflecting the uneven distribution of the rehabilitation workforce and the historical concentration of services in hospital and urban settings (8, 19–21). Systemic fragmentation reflects enduring governance and organisational failures, including weak coordination mechanisms, the inadequate integration of rehabilitation into health services, and chronic underinvestment. These limitations undermine continuity of care and disproportionately affect people with chronic conditions and long-term functional limitations, who require sustained rehabilitation over time. Territorial inequities further exacerbate these challenges, as the concentration of rehabilitation resources in hospitals and urban areas restricts access for rural populations and those who are socioeconomically disadvantaged (15, 22). Together, these structural constraints demonstrate that fragmentation is deeply rooted in policy priorities and service design choices, not merely operational.

### 2.2 Community-based rehabilitation as an integrated and sustainable response

Community-based rehabilitation has emerged as a key response to these challenges, aligned with principles of integrated and person-centred care. Evidence suggests that rehabilitation approaches embedded in the community and articulated with primary health care and social support networks are associated with improved functional outcomes, enhanced self-management and greater user satisfaction (18, 20). By shifting the focus from episodic interventions to sustained support within everyday living contexts, community-based rehabilitation reframes rehabilitation as a continuous and adaptive process (2, 13).

**Figure 1:**
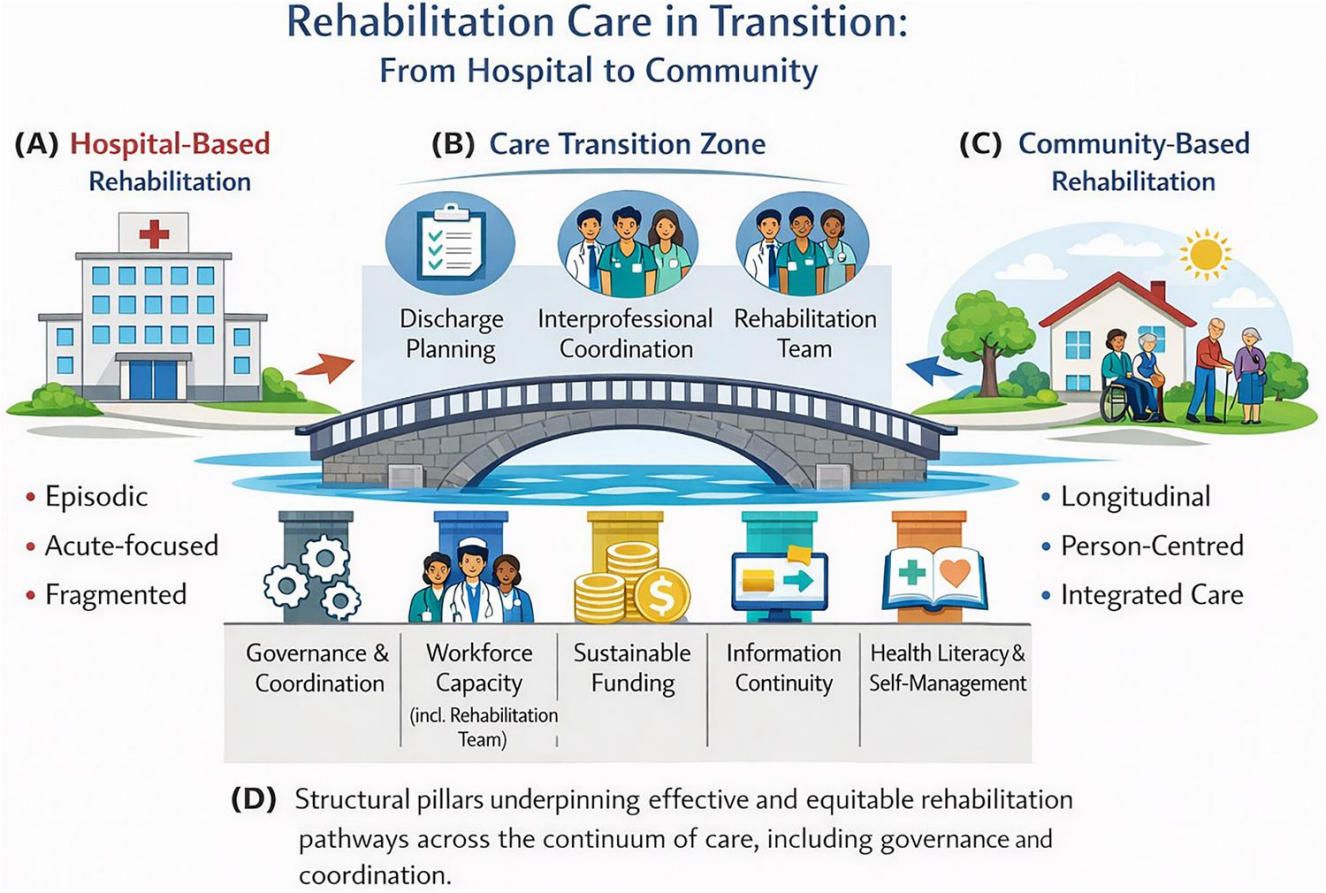
A conceptual framework of rehabilitation care in transition from hospital-based to community-based rehabilitation. **(A)** Hospital-based rehabilitation, characterised by episodic, acute-focused and frequently fragmented care. **(B)** Care transition zone, representing the critical interface between hospital and community settings, emphasising discharge planning, interprofessional coordination and the role of the rehabilitation team in ensuring continuity of care. **(C)** Community-based rehabilitation, illustrating a longitudinal, person-centred and integrated approach embedded in everyday living contexts. **(D)** Structural pillars underpinning effective and equitable rehabilitation pathways across the continuum of care, including governance and coordination, workforce capacity (including the rehabilitation team), sustainable funding, information continuity, and health literacy and self-management support. *Source: Authors' own elaboration, informed by international rehabilitation and integrated care frameworks*.

To synthesise these perspectives, we propose a conceptual framework of rehabilitation care in transition (Figure 1). Informed by international rehabilitation and integrated care frameworks (2, 23), this conceptual framework, the model conceptualises rehabilitation as a continuous pathway across the care spectrum, rather than as an endpoint of discharge. Hospital-based rehabilitation is represented as an initial, acute-focused, time-limited phase. In contrast, community-based rehabilitation sustains, adapts and consolidates functional gains over time within people's everyday living contexts. The transition zone between these settings is explicitly highlighted as a critical interface for discharge planning, interprofessional coordination and ensuring continuity of care by rehabilitation teams.

The conceptual framework further identifies governance and coordination, workforce capacity, sustainable funding, information continuity and support for health literacy and self-management as key structural pillars underpinning effective and equitable rehabilitation pathways (2, 21). By integrating these elements, we argued that community-based rehabilitation is positioned not merely as a service reconfiguration, but as a strategic investment that promotes functional capacity, autonomy and social participation as public health outcomes (3, 21).

## 3 Health literacy as a driver of empowerment

Health literacy is now recognised as a key determinant of health outcomes, transcending the individual dimension to establish itself as a structuring resource for healthcare systems. In community rehabilitation, it plays a particularly strategic role, enabling clinical interventions to translate into sustained self-care, therapeutic adherence and active participation, integrated into people's daily lives.

### 3.1 Health literacy and rehabilitation processes

Health literacy is defined by the ability to access, understand, evaluate and apply health information to make informed decisions (24). This concept involves not only understanding, but also critical thinking, effective interaction with professionals and autonomy in health management (11). Empirical evidence shows that higher levels of health literacy are associated with better rehabilitation outcomes, including functional gains, greater adherence and better perception of health status (25, 26).

It is important to note that this relationship is bidirectional: rehabilitation interventions can themselves enhance health literacy by strengthening self-management skills and confidence throughout the therapeutic process (27).

### 3.2 Health literacy, equity and community intervention

Health literacy also plays a mediating role in inequalities in access to and benefit from rehabilitation. Recent evidence from the German lidA cohort shows that the association between lower educational attainment and greater unmet subjective need for rehabilitation is partially mediated by lower levels of health literacy, suggesting difficulties in understanding available interventions and navigating health services (28).

Interventions targeting health literacy have a positive impact, especially when they adopt simple, multimodal, and person-centred approaches. Strategies such as plain language, visual materials, teach-back techniques, and digital literacy support improve adherence, self-efficacy, and integration of therapeutic recommendations into daily life (29, 30).

In order to operationalise health literacy in community rehabilitation, it must be systematically integrated into clinical practice and service design. Rehabilitation professionals must adopt communication strategies such as plain language, teach-back techniques and visual or multimodal educational materials tailored to individuals' capabilities and contexts. At an organisational level, literacy-sensitive service pathways, co-designed educational resources and digital literacy support are essential to ensure that rehabilitation interventions lead to sustained self-management and participation. Integrating health literacy as a core competency within rehabilitation teams empowers individuals and prevents the perpetuation of social and health inequalities.

## 4 Digital transformation and integration of care

Digital transformation has the potential to make rehabilitation more accessible, strengthen therapeutic continuity and improve coordination between hospitals, primary care and community-based services. The introduction of the European Health Data Space (EHDS) in March 2025 will establish a European framework for securely sharing health data. This will promote cross-border access and citizens' control over their data, as well as the reuse of data for research and innovation. This will have a direct impact on care integration (31).

There is consistent evidence from several musculoskeletal and post-operative conditions that telerehabilitation is non-inferior to face-to-face interventions, with comparable gains in pain and function, and it is potentially cost-effective. Recent systematic reviews and meta-analyses support the use of telerehabilitation as a complementary or hybrid model, particularly in contexts characterised by geographical, organisational or logistical barriers (32, 33).

At an organisational level, interoperability between electronic health records and health information exchange (HIE) systems has been shown to improve the safety, continuity and efficiency of care. This reduces redundancies and failures during transitions, which are critical aspects for integrated rehabilitation trajectories (34, 35). Combining interoperable digital tools with community-based care models can further enhance coordination, shared learning, and responsiveness at the territorial level.

Despite its potential, digital transformation in rehabilitation faces significant challenges. Governance and regulatory frameworks must ensure the ethical use and equitable access of data, particularly in the context of expanding infrastructures for data sharing, such as the EHDS. Organisational readiness, interoperability limitations and uneven digital competencies among health professionals are persistent barriers across health systems. Furthermore, usability constraints and lower levels of eHealth literacy among older adults and socioeconomically disadvantaged groups could restrict access to and adherence with digital rehabilitation solutions, potentially exacerbating existing inequalities if digital interventions are not user-centred and accompanied by empowerment strategies (36, 37).

Integrated community care models such as Buurtzorg illustrate how articulating relational care approaches with simple, interoperable digital tools can improve satisfaction and coordination while reducing avoidable hospitalisations. However, the economic effects remain context-dependent (38, 39).

Taken together, these experiences suggest that effective care integration relies on aligning three interdependent components: telerehabilitation as a service delivery modality, digital interoperability as an enabling infrastructure, and community-based care models as an organisational foundation. Within this framework, EHDS provides the regulatory backbone, clinical evidence supports telerehabilitation, and the digital empowerment of citizens ultimately determines whether digital transformation becomes truly inclusive and person-centred.

## 5 Discussion and future perspectives

The proposed conceptual model (Figure 1) provides an analytical framework for understanding the transition from fragmentation to empowerment in contemporary health systems. Rather than depicting a linear service pathway, the model represents an integrated space of empowerment that emerges at the intersection of community rehabilitation, health literacy and digital transformation. Community rehabilitation ensures functional continuity and social participation, while health literacy enables individuals to translate knowledge into informed action and shared decision-making. Meanwhile, digital transformation connects professionals, services, and life contexts across time and space. Together, these dimensions transform individuals from passive recipients of care into active co-producers of health and functionality.

The intersection of these three axes represents a qualitatively distinct space in which individuals cease to be passive recipients of care and become active co-producers of their health and functionality. There is strong international evidence supporting this integrative approach, recognising the integration of rehabilitation across the care pathway as essential for achieving sustainable health systems aligned with the Sustainable Development Goals (SDGs), particularly SDGs 3 and 10 (21, 40). The transition from fragmentation to empowerment is one of the most profound and necessary shifts facing contemporary health systems. This involves a technical reorganisation of services as well as a broader ethical, political and cultural paradigm shift that places individuals at the centre of care and redefines the role of rehabilitation throughout life, particularly in relation to chronic conditions and ageing. Within this framework, community rehabilitation, health literacy and digital transformation are not separate or optional domains, but rather interdependent pillars of more equitable, resilient and autonomy-oriented care systems.

From an operational perspective, implementing this model presents persistent challenges, including fragmentation between levels of care, chronic underfunding, territorial inequalities and limitations in health and digital literacy. However, these constraints coexist with significant structural opportunities. Expanding community- and home-based rehabilitation, developing interoperable information systems driven by European initiatives, growing telerehabilitation and investing in community empowerment programmes are all tangible ways to improve the continuity, efficiency and equity of care. Nevertheless, effective integration requires sustained clinical and political leadership, a redefinition of budget priorities, and consistent investment in professional training, particularly in health communication, literacy, and the ethical use of technology.

In this context, rehabilitation nurses play an indispensable strategic role. Their proximity to individuals, families, and communities, combined with their ability to coordinate care between hospital and community settings, positions them as pivotal figures in translating the principles of integrated care into practice. Evidence suggests that home-based programmes led by rehabilitation nurses can reduce readmissions, improve functional outcomes and enhance quality of life, thereby reinforcing the importance of community rehabilitation in modern healthcare systems (41).

From a research perspective, there is a clear need to develop integrated assessment models aligned with the International Classification of Functioning, Disability and Health (ICF) that can articulate clinical, functional, educational and technological outcomes (42). Economic evaluations of integrated rehabilitation and systematic assessments of the impact of health literacy and digital equity should occupy a central place in future research agendas to inform more robust, evidence-based and sustainable policy decisions.

Ultimately, community rehabilitation integrated with health literacy and digital transformation emerges as a structuring strategy for dignity, autonomy, and social cohesion. The transition from fragmentation to empowerment is not merely desirable, but a strategic necessity for health systems that aspire to be truly person-centred and capable of responding effectively to the demographic, social and technological challenges of the 21st century.

## Data Availability

The original contributions presented in the study are included in the article/supplementary material, further inquiries can be directed to the corresponding author.
